# Expression of RNAs Coding for Metal Transporters in Blood of Patients with Huntington’s Disease

**DOI:** 10.1007/s11064-015-1737-4

**Published:** 2015-10-15

**Authors:** Monika Szeliga, Aleksandra Różycka, Paulina Jędrak, Sylwia Barańska, Piotr Janik, Zygmunt Jamrozik, Jan Albrecht

**Affiliations:** Department of Neurotoxicology, Mossakowski Medical Research Centre Polish Academy of Sciences, 5 Pawińskiego Str., 02-106 Warsaw, Poland; Faculty of Horticulture, Biotechnology and Landscape Architecture, Warsaw University of Life Sciences, 166 Nowoursynowska Str., 02-787 Warsaw, Poland; Department of Molecular Biology, University of Gdańsk, 59 Wita Stwosza Str., 80-308 Gdańsk, Poland; Department of Neurology, The Wolski Hospital im Dr Anny Gostyńskiej, 17 Kasprzaka Str., 01-211 Warsaw, Poland; Department of Neurology, Medical University of Warsaw, 1 Banacha Str., 02-097 Warsaw, Poland

**Keywords:** Huntington’s disease, Metal transporter, TF, TFR, DMT1, ZIP8

## Abstract

Recent studies have demonstrated elevated levels of iron (Fe) in brains of patients with Huntington’s disease (HD). Striatal cells carrying mutated *Huntingtin* presented increased sensitivity to cadmium (Cd) toxicity, decreased sensitivity to manganese (Mn) toxicity and deficits in Mn uptake. The hypothesis arose that the observed alterations result from the altered expression and/or activity of proteins engaged in the transport of these metals, that is: transferrin (TF), transferrin receptor (TFR), divalent metal transporter 1 (DMT1) and ZIP8 protein. Here we examined the expression levels of genes encoding these proteins in blood of HD patients and control subjects. A decreasing tendency in the level of *TF* transcript and increasing tendency of *SLC11A2* mRNA encoding DMT1 was observed in the blood of HD patients compared to the control subjects, but neither attained statistical significance. No changes were found in the levels of *TFRC* coding for TFR and *SLC39A8* coding for ZIP8 between HD patients and controls. The results indicate that HD-associated changes in metal homeostasis occur are not related to mechanisms other than the expression level of the here analyzed metal transporters.

## Introduction

Huntington’s disease (HD) is a chronic and progressive neurodegenerative disease clinically characterized by chorea, psychiatric, psychological and intellectual disorders, and neuropathologically by the loss of striatal projection neurons [1]. Currently, there is no effective treatment to delay the onset or significantly slow the progression of HD. HD is an autosomal-dominant disorder caused by mutation of the *IT15* gene (also known as *HTT*) encoding huntingtin (Htt). The detailed role of wild-type Htt is unclear, although a growing body of studies indicates its function in brain development and embryogenesis [2], autophagy [3], and regulation of gene transcription [4, 5]. It also protects against cell death and has anti-apoptotic properties [6, 7]. Huntingtin knockout mice exhibit embryonic lethality [8]. Wild-type human Htt contains the polyQ domain composed of 11–34 glutamine (CAG) residues. HD is caused by an abnormal (>35) expansion of the CAG repeats [1]. Proteolysis of mutant Htt (mHtt) releases multiple N-terminal Htt fragments containing expanded polyQ repeats which aggregate in nucleus and cytoplasm of affected neurons [9]. It is still unclear whether the aggregates are cytotoxic *per se*, although the cytotoxic effect of mHtt have been documented in distinct HD models [10]. Recent findings identified several proteins interacting with mHtt which may contribute to the pathology of HD [9].

Lack of correlation between the selective degeneration and widespread expression of mHtt strongly suggests that other factors may increase the vulnerability of striatal neurons to the pathophysiological mechanisms underlying HD [11, 12]. Growing evidence links HD to altered metal homeostasis. Increased levels of iron (Fe) and copper (Cu) were found in postmortem brain tissue from patients with HD compared with control subjects [13–15]. Studies on a mutant STH*dh*^*Q111*/*Q111*^ cell line, a striatal neuronal cell line model of HD, revealed changes in Fe signaling and elevated level of transferrin receptor (TfR) in comparison with the wild-type STH*dh*^*Q7*/*Q7*^ cells [16]. The mutant STH*dh*^*Q111*/*Q111*^ cells displayed an increased sensitivity to cadmium (Cd) toxicity and resistance to manganese (Mn) toxicity [17]. Of note, this neuroprotective interaction was highly metal specific. Moreover, a decreased accumulation of Mn was observed in the mutant STH*dh*^*Q111*/*Q111*^ cells and in the YAC128Q HD mouse model [17]. The hypothesis arose that the observed alterations result from the altered expression of proteins engaged in the transport of heavy metals, that is: divalent metal transporter 1 (DMT1), ZIP8 protein, transferrin (TF) and transferrin receptor, TfR [19]. As a step towards verifying the hypothesis, we compared the expression levels of genes encoding these proteins in blood of HD patients and control subjects.

## Materials and Methods

### Participants

Fifteen HD patients were recruited from the Department of Neurology, Medical University of Warsaw, Warsaw, Poland (seven patients) and the Specialist Hospital św. Wojciecha in Gdańsk, Poland (eight patients). All patients were positive on the molecular test for the presence of a CAG triplet >35 repeats in the *HTT* gene. They also manifested clinical signs and symptoms of HD. Clinical examination was performed by trained [formal and certified training within the European Huntington’s Disease Network (EHDN)] neurologists and psychologists and included: motor and behavioural rating due to Unified Huntington’s Disease Rating Scale (UHDRS) and neuropsychological rating for depression (Hamilton and Beck’s questionnaires), TFC (Total Functioning Capacity) and cognitive assessment (Stroop Test, Verbal Fluency Test, Symbol Digit Modality Test). A control group (age and gender matched) included volunteers with no neurodegenerative disorders. All of the participants received verbal and written information about the study, and signed an informed consent form. The local Ethics Committees approved all procedures. Table 1 shows the subjects’ gender, age and the length of the CAG repeat on the expanded allele.

**Table 1:** Clinical and genetic characteristics of HD patients and healthy controls

Samples	No.	Sex (women/men)	Age (years)	CAG size
Control	15	9/6	46 (27–70)	–
HD	15	9/6	57 (33–78)	41 (39–51)

Age and CAG size are expressed as median values (min–max). The number of CAG repeats is missing for 4 HD patients

### RNA Extraction

Peripheral blood lymphocytes were isolated from blood of patients and control subjects from the Medical University of Warsaw, Poland. First, the blood sample was diluted with an equal volume of phosphate buffered saline (PBS) and poured onto the Ficoll solution (Sigma-Aldrich). The test tube was centrifuged for 20 min at 1600 rpm. The ring with white blood cells was harvested and washed twist with PBS. Next, total RNA was extracted from the lymphocytes using TRI Reagent BD (Sigma-Aldrich), according to the manufacturer’s protocol. Total RNA was extracted from whole blood of patients and control subjects from the Specialist Hospital św. Wojciecha in Gdańsk, Poland, using PAXgene Blood RNA Kit (Qiagen) according to the manufacturer’s protocol. Total RNA concentration was measured with a NanoDrop 1000 Spectrophotometer.

### Reverse Transcription and Real-Time PCR

One microgram of RNA was digested with DNaseI (Invitrogen) and reverse-transcribed using a High-Capacity cDNA Reverse Transcription Kit (Applied Biosystems) according to the manufacturer’s protocol. Two independent reverse transcription reactions were performed for every RNA sample. Real-time PCR was performed using TaqMan Gene Expression Assays listed in Table 2 and TaqMan Universal PCR Master Mix (Applied Biosystems) according to the manufacturer’s protocol. The reactions were incubated at 95 °C for 10 min, followed by 45 cycles of 95 °C for 15 s and 60 °C for 1 min using an Applied Biosystems 7500 Sequence Detection System. Relative expression was normalized to the expression of *ACTB* gene encoding β-actin and calculated using the ΔΔC_T_ method [20].

**Table 2:** TaqMan gene expression assays used for real-time PCR

Transcript	Assay ID	GeneBank sequence	Exon boundary
TF	Hs01067777_m1	AK222755.1	14–15
TFRC	Hs00951083_m1	AB209254.1	16–17
SLC11A2	Hs00167206_m1	AB004857.1	15–16
SLC39A8	Hs00223357_m1	AB040120.1	3–4
ACTB	4326315E	NM_001101.2	1

### Statistical Analyses

The data were analyzed using the paired Student’s *t* test. In the box plots, the boundary of the box closest to zero indicates the 25^th^ percentile, the line in the middle is plotted at the median, and the boundary of the box farthest from zero indicates the 75^th^ percentile. The Pearson’s correlation coefficient was used to identify correlations between the number of CAG repeats and the level of *SLC11A2* and *TF* mRNA levels normalized to *ACTB* mRNA.

## Results

**Fig. 1:**
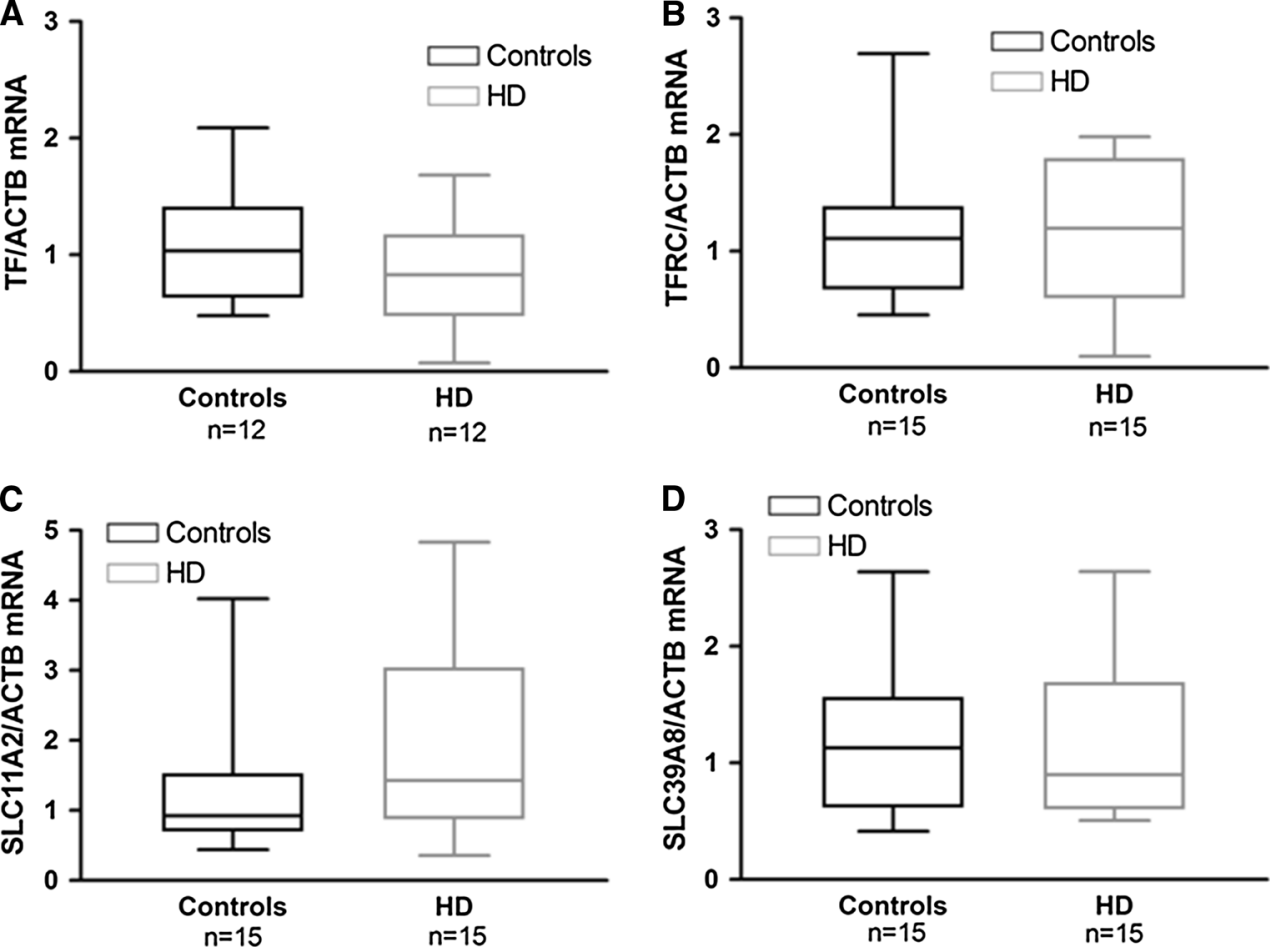
Relative levels of manganese transporters transcripts in blood from control and HD subjects. Levels of *TF* (**a**), *TFRC* (**b**), *SLC11A2* (**c**) and *SLC39A8* (**d**) mRNA were normalized to beta-actin (ACTB). HD, HD patients. The *boundary* of the *box* closest to zero indicates the 25^th^ percentile, the *line* in the *middle* is *plotted* at the median, and the *boundary* of the *box* farthest from zero indicates the 75^th^ percentile

We performed real-time PCR analysis of expression of *TF*, *TFRC*, *SLC11A2* (coding for DMT1) and *SLC39A8* (coding for ZIP8) genes in blood of HD and control subjects. A statistically insignificant decreasing tendency in *TF* mRNA level was observed in HD patients (mean value: 0.8369; median value: 0.8285) compared to controls (mean value: 1.072; median value: 1.034) (Fig. 1a). Of note, *TF* mRNA was undetectable in 3 out of 15 HD patients, therefore the analysis of *TF* expression was performed on 12 HD and 12 healthy subjects. No difference was found in *TFRC* mRNA level between controls (mean value: 1.080; median value: 1.109) and HD patients (mean value: 1.124; median value: 1.197) (Fig. 1b). *SLC11A2* mRNA showed a tendency to increase in HD patients (mean value: 1.748; median value: 1.428) with respect to controls (mean value: 1.259; median value: 0.9238), although this difference was not statistically significant (*p* > 0.05; Fig. 1c). There was no statistically significant difference between the level of *SLC39A8* transcript in controls (mean value: 1.174; median value: 1.129) and HD patients (mean value: 1.133; median value: 0.8990) (Fig. 1d).

**Fig. 2:**
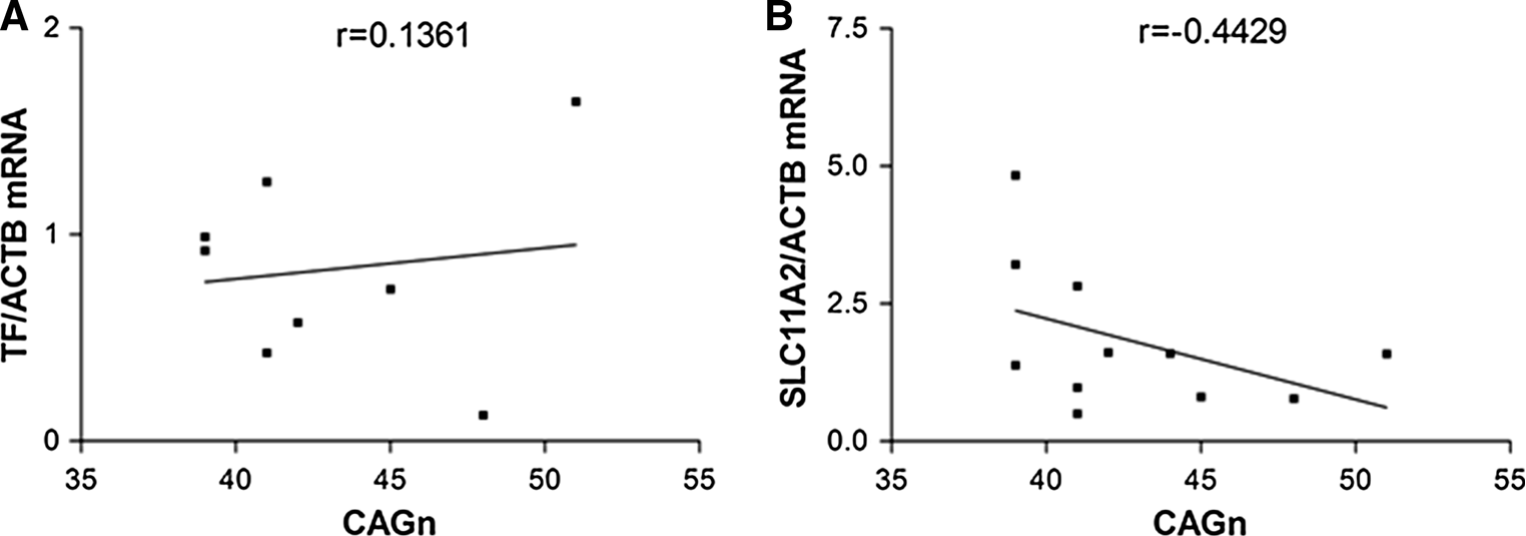
Lack of correlation between the numbers of CAG repeats and the relative level of *TF* (**a**) or *SLC11A2* (**b**) mRNA in blood of HD patients. As data concerning the number of CAG repeats were missing for 4 HD patients, the analysis was performed on 8 samples in case of *TF* mRNA (**a**) and 11 samples in case of *SLC11A2* mRNA (**b**)

Next we examined whether the alterations in *TF* and *SLC11A2* mRNA levels observed in blood of HD patients were associated with the number of CAG repeats. As data concerning CAG size were missing for 4 samples, this analysis was performed on 8 and 11 samples in the case of *TF* and *SLC11A2* mRNA, respectively. We found no correlation between the number of CAG repeats and the level of *TF* (r = 0.1361, *P* = 0.7480, Pearson’s test) or *SLC11A2* (r = −0.4429, *P* = 0.1725) mRNA (Fig. 2a, b, respectively).

## Discussion

Fe and Mn play roles in a number of physiological processes, yet the exposure to excessive levels of these metals can cause a damage to the nervous system. Association between altered homeostasis of these metal and neurodegenerative disorders, including HD, has been frequently postulated (for extensive reviews, please refer to [21–24]). Alterations in Fe signaling were observed in a mutant STH*dh*^*Q111*/*Q111*^ cell line, a striatal neuronal cell line model of HD as compared to the wild-type STH*dh*^*Q7*/*Q7*^ cells [16]. Increased level of Fe was found in R6/2 HD mice [25] and in brain tissue from HD patients [13–15]. Recent studies revealed increased sensitivity of STH*dh*^*Q111*/*Q111*^ cell line to Cd toxicity and resistance to Mn toxicity. Moreover, the same studies showed a decreased Mn accumulation in STH*dh*^*Q111*/*Q111*^ cells and YAC128Q HD mice following in vivo Mn exposure [17, 18].

Here we addressed the hypothesis that disturbed Fe and Mn homeostasis observed in HD models are caused by changes in the function of metal-transporting proteins and we asked the question whether this pathogenetic pattern also holds for human HD. As a step toward this end, we analysed the expression of genes encoding the four proteins most evidently involved in the transport of these metals and tightly link Fe and Mn homeostasis to each other. A growing body of evidence suggests that transferrin (TF), the main Fe-carrying protein in neurons [26] and transferring receptor (TFR) play crucial role in brain Mn transport [19, 27, 28]. STH*dh*^*Q111*/*Q111*^ cells exhibit alterations in the level of TFR protein: its decreased level was found in the early passages of this cell line [16], while the late passages presented an increased level of this protein [18] compared with a wild-type cells STH*dh*^*Q7*/*Q7*^. While our data demonstrate a decreasing tendency in the level of *TF* transcript in the blood of HD patients compared to the control subjects, no correlation was observed between the relative level of *TF* transcript and the numbers of CAG repeats, a hallmark of HD. We have also observed lack of differences in the level of *TFRC* transcript between HD patients and controls.

Another protein involved in Fe and Mn delivery is DMT1 (divalent metal transporter-1, also known as NRAMP-2) [29–31]. This protein is of interest as it has been implicated in the transfer of Mn and other metals across the blood–brain barrier [32]. Dysfunction of DMT1 has been linked to many disorders including Parkinson’s [33] and Alzheimer’s [34] disease. A slight trend toward increased DMT1 level was observed in STH*dh*^*Q111*/*Q111*^ cells compared with the wild-type cells [18]. In our study, *SLC11A2* mRNA encoding DMT1 showed a tendency to increase in HD patients compared with controls. However, as was the case with the *TF* transcript, the increase did not reach statistical significance and the slight changes did not correlate with the number of CAG repeats. It will have to be verified whether the tendencies towards decrease/increase noted above on the limited number of samples will reach statistical significance when larger amounts of material become available.

We have also examined the expression of *SLC39A8* encoding ZIP8, as this protein is involved in Fe transport in hippocampal neurons [35] and some findings suggest its role in Mn transport [36, 37]. A point mutation p.A391T in *SLC39A8* has been associated with some disorders including schizophrenia [38]. We have found no differences in the level of *SLC39A8* transcript between the blood of HD and control subjects.

In conclusion, our study failed to demonstrate changes in the expression level of genes coding for the major brain transporters of Fe and Mn: TF, TFR, DMT1 and ZIP8 between blood of HD patients and controls. If impaired metal homeostasis in human HD is related to the dysfunction of the group of metal transporters here analyzed, the underlying mechanisms will have to involve one or several steps post transcription. On the other hand, evidence has recently begun to accumulate suggesting a role of efflux and efflux-mediating proteins in maintaining metal homeostasis (for an exhaustive review see Ref. [32]). The absence of changes in the expression of metal transporters dealt with in the present study does not exclude contribution of other transporting moieties that have attracted attention more recently, such as ZIP14, DAT, citrate transporters, Ca channels, SLC30A10 and ferroportin (for an exhaustive review see Ref. [32]). Most significantly, hypermanganesemia with associated Parkinson Disease symptoms has been found related to mutations in the *SLC30A10*, a member of the SLC30 solute carrier subfamily previously thought to serve as a specific Zn transporter [39, 40]. In this context, analysis of the expression and sequence of RNAs coding metal efflux-mediating proteins in HD patients appears to be an interesting venue of further investigations.

It may be argued that the results of gene expression obtained from blood analysis may not be unconditionally representative for the expression patterns of metal transporters in the brain, a question that we were unable to attend practically. However, numerous recent reports have convincingly documented a good correlation between the brain tissue and peripheral readouts of other genes purportedly associated with the development of HD. Positive correlation in this regard has been noted, for instance, for TGF-β1 [41] and Rho kinase pathway genes [42]. Clearly, the present results will have to be verified once access to representative brain tissue is gained.

Irrespective of the above considerations, it appears mandatory to use a functional approach, starting with comparing metal transport in lymphocytes derived from control and HD subjects.
